# Simulation educators in clinical work: the manager's perspective

**DOI:** 10.1108/JHOM-04-2018-0107

**Published:** 2020-02-19

**Authors:** Éva Tamás, Marie-Louise Södersved Källestedt, Håkan Hult, Liisa Carlzon, Klas Karlgren, Magnus Berndtzon, Magnus Hultin, Italo Masiello, Renée Allvin

**Affiliations:** 1 Institution for Medicine and Health, Linkopings Universitet, Linkoping, Sweden; 2 Clinical Skills Centre, Centre for Clinical Research, Uppsala University, Uppsala, Sweden; 3 Department of Clinical Science Intervention and Technology, Karolinska Institutet, Stockholm, Sweden; 4 Simulation Centre West, Department of Research, Education and Development, Sahlgrenska University Hospital, Goteborg, Sweden; 5 The Södersjukhuset Hospital and Department of Learning, Informatics, Management and Ethics, Karolinska Institutet, Stockholm, Sweden; 6 Metodikum – Skill Centre of Medical Simulation, Region County Jönköping, Jönköping, Sweden; 7 Department of Surgical and Perioperative Sciences, Anaesthesiology and Intensive Care (Sunderbyn), Umeå University, Umeå, Sweden; 8 Department of Clinical Science and Education, Karolinska Institutet, Stockholm, Sweden; 9 Clinical Skills Centre, Faculty of Medicine and Health, School of Medical Sciences, Örebro University, Örebro, Sweden

**Keywords:** Organisational learning, Patient safety, Continuing medical education, Patient simulation, Community of practice, Teamwork

## Abstract

**Purpose:**

Information is scarce on healthcare managers' understanding of simulation educators' impact on clinical work. Therefore, the aim of this study was to explore healthcare managers' perceptions of the significance of clinically active simulation educators for the organisation.

**Design/methodology/approach:**

Healthcare managers were invited to be interviewed in a semi-structured manner. Inductive thematic analysis was used to identify and analyse patterns of notions describing the managers' perceptions of simulation educators' impact as co-workers on their healthcare organisations.

**Findings:**

The identified relevant themes for the healthcare unit were: (1) value for the manager, (2) value for the community and (3) boundaries. Simulation educators were perceived to be valuable gatekeepers of evidence-based knowledge and partners in leadership for educational issues. Their most prominent value for the community was establishing a reflective climate, facilitating open communication and thereby improving the efficacy of teamwork. Local tradition, economy, logistics and staffing of the unit during simulation training were suggested to have possible negative impacts on simulation educators' work.

**Practical implications:**

The findings might have implications for the implementation and support of simulation training programs.

**Social implications:**

Healthcare managers appreciated both the personal value of simulation educators and the effect of their work for their own unit. Local values were prioritised versus global. Simulation training was valued as an educational tool for continual professional development, although during the interviews, the managers did not indicate the importance of employment of pedagogically competent and experienced staff.

**Originality/value:**

The study provided new insights about how simulation educators as team members affect clinical practice.

## Introduction

Hospital mortality due to professionals' error ( Kohn, 2000 ; Vincent *et al.* , 2001 ) can be prevented by regular medical simulation training. For the last two decades, continuing professional education has been introduced as part of continuing professional education to better prepare healthcare professionals for safer patient management.

Medical simulation focuses on individual and organisational learning and also affects patient care ( Reich *et al.* , 2015 ). The body of evidence for improving healthcare by using simulation is expanding ( Fritz *et al.* , 2017 ; Mahmoudian-Dehkordi and Sadat, 2017 ). Healthcare professionals acquire competence and confidence through simulating patient management and receiving training in technical and non-technical skills in established clinical simulation centres and *in situ* at healthcare units ( Mcpherson *et al.* , 2001 ; Shapiro *et al.* , 2004 ; Aggarwal *et al.* , 2010 ). Issenberg , (2006) identified three essential components of effective use of simulation: training resources, trained educators and curricular institutionalisation. A systematic educational intervention by medical simulation was found challenging from both economic and staffing perspectives ( Salas *et al.* , 2009 ; Salas and Rosen, 2013 ).

Professional practice is an arena for learning, but healthcare professionals often tend to consider it to involve only performing their duties rather than learning ( Boud and Solomon, 2003 ). Thus, the pedagogical process in many clinical situations is not something obviously planned or supported ( Hult *et al.* , 2009 ). However, working together can be the shared experience, which is the prerequisite for learning, and any given group of people sharing learning experiences can be defined as a community of practice ( Wenger, 1998 ). Healthcare professionals are usually involved in several communities of practice and share different goals, values and methods through time. Members of a community may have different and complementary work experiences at different levels.

Simulation educators (professionals facilitating medical simulation training sessions) usually work both clinically, as healthcare professionals, and at simulation training centres. Thus, they belong to several communities of practice at the same time. Consequently, they interact with and affect their colleagues both in simulated and in real-life environments. In a previous multi-centre study, experienced healthcare simulation educators were found to notice a development of professional competency based on their expanding confidence ( Allvin *et al.* , 2017 ).

Although healthcare managers are stake-holders, and simulation educators have a key-role for planning continuing professional educational interventions, there is no available information in the literature on how healthcare managers perceive simulation educators' impact on clinical practice, not just as educators but as co-workers. Therefore, the aim of the present study was to explore healthcare managers' perceptions of the significance of clinically active simulation educators for the operation of the organisation.

## Method

### Design and participants

In the present study, the healthcare managers, hereafter managers, in the community of practice where the previously studied educators ( Allvin *et al.* , 2017 ) worked as clinicians, were invited to participate. To obtain knowledge of the managers' perceptions of the simulation educators' significance for the operation of the clinical organisation, an explorative design with a qualitative approach ( Polit and Beck, 2011 ) was used. The managers were personally asked about participation in the study. Of 14 possible, 11 (six females and five males) accepted, one manager declined participation and two were not available. They represented different professional groups (eight registered nurses, two physicians and one midwife), with experience as manager ranging between 2 and 34 years (mean 11). They worked as mangers at different medical departments (obstetrics *n* = 1, paediatric *n* = 3, emergency unit *n* = 1, pre-hospital unit *n* = 1, intensive care unit *n* = 3, anaesthesiology *n* = 2) in seven different hospitals. The Regional Ethical Review Board of Linköping, Sweden, approved the study (ref: 2014/204-231).

### Data collection

The research team conducted individual face-to-face interviews ( Kvale and Brinkmann, 2009 ), each lasting between 18 and 40 minutes. Data were collected between December 2015 and October 2016 by one researcher at each simulation centre. All the researchers had a range of educational and working experience in terms of their simulation education background, and had worked both in simulation centres and in university medical and nursing education. Before the interview started, all interviewers agreed on common basis and pre-understanding.

The interview questions were open-ended and followed an interview guide to cover the central areas for the aim of the study. All interviews started with the same questions: ‘Can you please tell me about your understanding of simulation in healthcare?, followed by ‘What is your view on simulation in relation to your healthcare organisation?’ After the opening questions, the participants were asked to reflect on and describe their experiences of having experienced simulation educators among their staff. All interviews were audio-recorded, transcribed verbatim and anonymized.

### Data analysis

**Figure 1:**
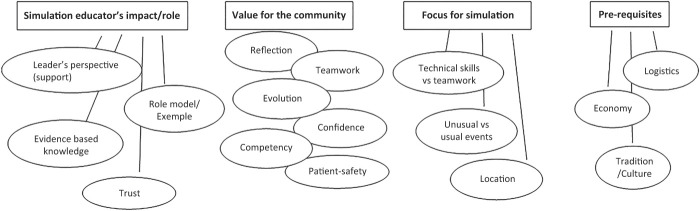
Preliminary thematic map. Following the initial analysis four main themes with loose groups of sub-themes were identified

**Figure 2:**
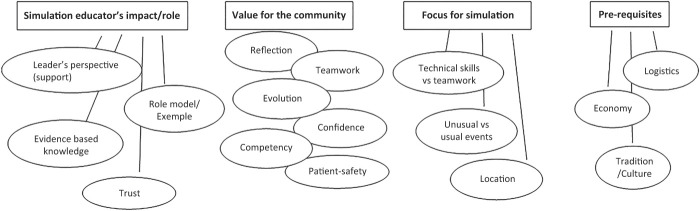
Final thematic map. The final map shows three main themes with respective sub-themes. After a first revision, there were still four themes without any hierarchy or other relationship identified among codes within themes at that stage, though one of the codes had been re-named. One of the previous main themes “Focus for simulation”, though present in each interview, was found to be an independent factor during the final analysis. Similarly, the code “Development” was found to be a common feature for several codes within the same theme rather than a stand-alone code. Therefore, these were eliminated

An inductive thematic analysis ( Braun and Clark, 2006 ) was used to identify and analyse patterns (themes) describing the managers' perceptions of having experienced simulation educators' working as clinicians in their healthcare organisations. In a first step, all the interviews were read and re-read as whole entities to obtain familiarity with all aspects of the data. The interviews were read by all authors individually, and then discussed in the whole research group. Ideas or patterns of perceptions relevant to the study were marked in the text, and ideas about what was in the data were written down. The second step involved identifying and coding meaningful groups of text that referred to the managers' understanding of the simulation educators' significance for the operation of the organisation ( Figure 1 ). Thereafter, the different codes were discussed and collated into potential overarching themes and sub-themes. Finally, the themes were discussed and reviewed in relation to the coded groups of text and the entire data set. The specifics of each theme and the overall narrative were refined ( Figure 2 ). The analysis involved constantly moving back and forth between the entire data set, the coded meaningful groups of the text and the ongoing analysis of the data. During the entire analysis process, discussions among the researchers were conducted to strengthen the consistency of the findings.( Kitto *et al.* , 2008 ).

## Findings and discussion

Three distinct themes were identified: (1) *value for the manager,* (2) *value for the community and* (3) *boundaries* ( Figure 2 ). Themes and codes are presented in the following text, supported by quotations in italics.

### Value for the manager

A common pattern in the managers' views of simulation educators' role in the clinical practice was that the educators stood out as gatekeepers of evidence-based knowledge and as partners in leadership, rather than mere executors. For example, simulation educators were described as important partners in discussions about the aims and learning objectives of training at the units because they usually saw educational needs from a different perspective compared to the managers. Although it is always the manager of the unit who decides the subject of the simulation training, they do seek the simulation educators' input on the staff's knowledge gaps to eliminate possible risks for the patients. Simulation educators' deeper understanding of the educational needs at both individual and organisational levels was denoted by the managers as the ‘giraffe perspective’.

*‘… a simulation centre must obviously be based on existing guidelines. Then there is quality assurance ‘this is how we do it' … So, it is so important that the competence of the simulation educators is maintained and updated; and connected to a centre which is evidence-based, so to speak’.* (Manager 5)

*‘It is evidently a dialogue within a dialogue because it is also very important-instructors [simulation educators] have a very important role; we have given them quite a bit of space to see where the needs are’.* (Manager 10)

*‘They are very much like giraffes. They can see things from above. See the context and because of that they are committed to what they do; everything from staffing to how to work as a nurse-in-charge …’.* (Manager 5)

Generally, simulation educators were given a free hand in planning and executing simulations, legitimised by having the aforementioned special perspectives.

The managers felt that simulation educators acted based on a deeper understanding of the relevance and used evidence-based knowledge in the clinical practice for quality work. Having regular and standardised simulation training at the unit would be difficult without simulation educators applying evidence-based medicine. Having a pedagogical background seemed to be advantageous when simulation educators needed to be involved in team discussions or when leading a team in the clinical practice. They were characterised as possessing an academic way of thinking and a capability for long-term planning. Based on the aforementioned characteristics, simulation educators earned the trust of their managers who often emphasised that the simulation educator of their unit acted as a role model for co-workers of the unit, and they were trusted to step in as leaders in difficult clinical situations.

*‘…they could be ambassadors at the unit. A lot of knowledge is picked up in courses for simulation educators … I hope they will be good examples and ambassadors for a way of working and thinking also in the clinical environment, and I think they are’.* (Manager 11)

### Value for the community

Simulation educators created value for the community by establishing a reflective climate in general, facilitating open communication and thereby improving the efficacy of teamwork.

*‘…simulation has an important role in dealing with everyday phenomena because one is given the possibility to reflect on why we do things in a certain way or why we don't do them in a certain way …’.* (Manager 11)

Simulation educators facilitated improved communication during training sessions, and this was described as a key component of improved teamwork by the managers. As part of the communication training, simulation educators focused on helping the individuals to speak up when their competency could help in patient management and replacing hierarchy with a focus on patient care. Pressing team members to change attitudes towards other co-workers of the team during simulation had noticeable repercussions on the everyday work of the unit, and this was considered unquestionably positive.

*‘…this is extremely positive, and I see that it had an effect. For example, I mentioned earlier that we were having problems with crowded rooms in emergency situations. This has been changed …’.* (Manager 10)

*‘There is a lot of focus on communication too, on communication and on the team, so you feel more confident in their company too’.* (Manager 3)

*‘Yes, it is positive. And this way of communication has been appropriated in another context too. You see the significance of clear-cut communication especially in the operating room …’.* (Manager 10)

Furthermore, simulation training led to improved medical competence and self-confidence in team members. This self-confidence was considered to result in improved patient care, according to the managers' perceptions.

*‘… [simulation] is one of the most competence-boosting [tools] we have, I think; it sustains competency …’.* (Manager 3)

*‘… it is a completely different workplace when you feel that you can master everything you are exposed to, and it feels good to train before it happens in real-life. Because it happens every week’.* (Manager 1)

*‘You may say [that simulation] ensures self-confidence in the staff. That's how you can summarize it’.* (Manager 6)

*‘… you get self-confidence in your professional role when you have trained for it’.* (Manager 9)

The interviewed managers described a definite development over time where simulation and simulation educators' work resulted in a distinct improvement and changes in clinical practice. Simulations prepare the staff for critical clinical everyday situations. Simulation was compulsory at every unit, and the managers considered it obvious that it should be. They often said that simulation was part of the everyday operation of the unit, and it would be unimaginable not to have it. The most important value, as expressed by the managers, was its positive effect on patient safety. They described a distinct improvement in patient management for certain patient groups, for example, due to improved communication within the team and across different disciplines.

*‘…in the end, the main result is patient safety, as I see it anyway. I’m convinced that the patient is taken better care of than before, and that is our main goal…’.* (Manager 4)

### Boundaries

Local tradition and culture, economy and logistics are factors with possible negative impacts on simulation educators' work. Those who have higher positions in the staff hierarchy, for example, physicians or older staff members, seem to have difficulty in participating in simulation training sessions, even though their active participation is essential, especially for team training aiming at changing attitudes regarding communication.

*‘… [I] appreciate the difficulty for creating space for this simulation training; mostly this is a question about culture, and I think that this culture has been changed if I compare it to how I experienced it here at the beginning when I got involved with simulation activities. So, I think that there is more openness today…’.* (Manager 11)

However, logistics was still considered as an issue of importance.

*‘We do try to get all the staff together [for simulation training]. Then it can take a while before… we are quite a big group and there is more mobility nowadays which leads to increased turnover of the staff’.* (Manager 7)

*‘The younger the physicians, the easier it is. They are not so tied up in the clinical work or have plenty of other different assignments to take care of. This is a difficulty leading to the younger ones training while the seniors don* ' *t’.* (Manager 9)

The staffing of the unit, while part of the staff was engaged in simulation training, was a further difficulty identified by the managers. Generally, reimbursement for regular simulation training was considered challenging. Though there were only a few measurable end-points connected to simulation, it was considered to be a good investment anyway because of the need for future improvement of the unit.

*‘You have to dare e.g. to employ X% of the nurses as educators at the unit not only at the simulation centre. You must dare to accept that if we are to be good at this then time will have to be diverted from patient care or from other areas here and now. But this is going to be a benefit later…’.* (Manager 5)

*‘… if you feel that you have to balance your educational resources then it can happen that you must keep to your budget, and since simulation is expensive you choose another alternative’.* (Manager 8)

The identified themes described healthcare managers' perceptions of how simulation educators' work substantially affects the clinical organisation. Simulation educators were described as having a considerable clinical impact through using and channelling evidence-based knowledge both in simulation and in clinical settings. They were trusted with designing the educational development of the unit and being role models as competent and self-confident healthcare professionals. The substantial influence of the simulation educators materialised as values for the unit such as establishing a reflective environment, improving teamwork and increasing competency and confidence, which altogether led to the ultimate value of improved patient management at the unit. The managers focused strictly on learning objectives for the simulation trainings, based on the needs for the given unit and not the needs of the wider organisation. Reflections on values and cooperation were kept at the same level and not extended to the bigger organisation the unit was part of.

The theme of boundaries concerned factors limiting the managers' possibilities to plan for and to maintain regular medical simulations for the unit. Indirectly, these factors were believed to affect the clinical work of simulation educators.

### The manager's perspective

Simulation educators belong both to the community of practice of educators and to the healthcare professionals. They move constantly between these communities, which is a superb opportunity for brokering. Brokering means advocating for changes of practice of the community ( Wenger, 1998 ). Moving back and forth widens the perspective of simulation educators, and this is what was described by the managers as a ‘giraffe perspective’ in the interviews. Furthermore, simulation educators were labelled as brokers driving changes in the communities of practice. This role was previously described to be an important factor for organisational learning ( Antonacopoulou, 2006 ). Evidence-based medicine is the basis for the work of an educator in academic medicine and is inevitably applied in the clinical practice by a simulation educator. The use of evidence-based medicine is noted by the managers, and simulation educators were perceived as role models for the other members of the unit in the everyday practice. Working in the healthcare community also makes it possible for simulation educators to identify the educational needs of the community of healthcare practice. The managers expressed trust in the simulation educators' abilities to identify educational needs, to set adequate learning objectives for simulation activities and to serve as examples for other members of the unit. However, there could be a gap between practical teaching experience and integration of learning theories during simulation training, as previous research has demonstrated ( Allvin *et al.* , 2017 ).

### The community's perspective

The learning of the individual member and sharing the knowledge among the members, very often across professions, are keystones for the very existence and the development for the practice of the community. Heaven *et al.* found that following communication skills training, only those clinical nurse specialists who received additional support during clinical work transferred the trained skills to the workplace ( Heaven *et al.* , 2006 ). This is in alignment with our findings that simulation educators, when working as healthcare providers, facilitate the transfer of newly acquired skills for the other members of their community of practice.

Simulation educators facilitate and influence pedagogical and clinical processes during simulation sessions. Their work was generally perceived to result in a reflective climate and improved communication, both at individual and team levels. Reflection, in practice, especially within medicine and healthcare, is considered to be a basic characteristic of a professional ( Schön, 1983 ). Reflective teams have a disposition to negotiate and renegotiate the meaning of the actual practice of the community (e.g., clinical routines of the unit), providing a positive spiral of progression.

Simulation training, often referred to as training or just ‘doing the job’ ( Boud and Solomon, 2003 ), was declared unanimously by the interviewed managers to improve the competency and self-confidence of the staff. The apparently unambiguous progression to becoming a competent and confident member of the community ultimately translated into the clinical routine as improved patient safety, and patient safety represented the utmost value for the healthcare units.

Interestingly, the interviewed managers focused strictly on their own community of practice, that is, only on their own unit. The managers' views reflect how profoundly delineated and well defined the actual community of practice is, despite being part of a larger organisational unit (e.g., a hospital). The interviews revealed an apparent lack of reflection about values created beyond the boundaries of the managers' own unit within the larger organisation. There is no evidence in the present material to support the idea that either this aspect was implicit or it was disregarded by the managers.

### Boundaries

The culture of a community of practice is mainly a question of shared values and goals ( Wenger, 1998 ). It is the leaders who establish these values and goals by setting up goals and reinforcing behaviours in alignment with these values. We found that the interviewed managers often delegated partly or entirely this task to the simulation educators. This trust, however, might result in non-participatory behaviour of the members of community. Although the required behaviour is full participation, certain professions chose non-participation on a regular basis. The reasons for non-participation might be to do with identity (positions in the hierarchy, fear of being seen as incompetent, etc.) or prioritisation of the practice (burden of production, lack of time) ( Wenger, 1998 ). The managers expressed frustration about these issues, but seemed to lack the means (financial and/or organisational support) to implement long-term changes. This corroborates the complexity and relevance of reflections about funding ( Festa *et al.* , 2017 ). Salas *et al.* described seven critical factors for successful team training ( Salas *et al.* , 2009 ). The ‘economy’ and ‘logistics’ sub-themes of the ‘boundaries’ theme connect to the success factor described as ‘determine required resources and time commitment and ensure their availability’. Despite ambitions of regular and repeated simulation activities, the pressure of production from the larger organisation within which the healthcare unit operates is substantial and perceived to be a strong limiting factor, just like economic restrictions. Maintaining steady practice in simulation-based training was described to be challenging due to the need for simultaneous staffing to ensure patient care and education, putting patient safety at risk.

### Strengths and limitations of the study

The interviews were conducted at seven different hospitals in Sweden. This needs to be taken into consideration when translating the findings to contexts in other countries. Several researchers performed the interviews, but great effort was put into discussing the questions and agreeing on a common basis and pre-understanding before the interviewing started. The choice of the interviewed managers was based on having a staff of simulation educators with long experience.

In the analysis, continuous discussions about themes and patterns in the findings were held between all the authors. The authors have different expertise, which gives different perspectives and opportunities for analytical discussions of the findings. To increase trustworthiness of the study, we strived for detailed descriptions of the study context, selection, data collection and analysis. The findings are illustrated with appropriate quotes to further enhance trustworthiness.

## Conclusions

Simulation educators were perceived to contribute with evidence-based knowledge and leadership for educational issues within their clinical environment. The most prominent value for their work was establishing a reflective climate, facilitating open communication and thereby improving the efficacy of teamwork even in the clinical practice, and not just during simulation sessions. Ultimately, according to the managers' perceptions, having an experienced simulation educator active in clinical environment resulted in improved patient safety.
